# Supplementary material for: The prevalence and patterns of exposure to interpersonal violence among men and women in Estonia
Source: PLoS One. 2020 Aug 14;15(8):e0237562. doi: 10.1371/journal.pone.0237562 (PMC7428354; doi:10.1371/journal.pone.0237562)
Supplement: Attachment — Submitted filename: PONE-D-19-24414_review.docx [file pone.0237562.s002.docx]

**Summary**

The goal of this study was to describe patterns in interpersonal violence among men and women in Estonia and to determine correlates of exposure to one or more forms of interpersonal violence. The authors successfully demonstrated that there exists considerable heterogeneity in interpersonal violence between men and women. However, it is not clear what this study additionally provides to the literature on violence. As such, I recommend that this paper be rejected for the reasons listed below

**Major Issues**

Introduction:

1. In this paper, the authors make an implicit assumption that family/intimate partner violence and community violence can be aggregated together. A discussion of why this is feasible is missing.
2. Relatedly, it is unclear why the authors chose to distinguish the three different forms of violence – physical, sexual, and emotional (yet chose to aggregate interpersonal violence).
3. A discussion of the hypothesized prevalence of violence (e.g. based on other studies in the same region, or IPV studies in Estonia) is missing.

Methods:

4. It is unclear why the aim is modified to “to analyze the association between PVA and variables identified in the literature to be associated with a higher risk for exposure to ***IPV among women***” when the introduction sets the paper up to avoid focusing solely on IPV among women. As such, the “independent variables” sub-section needs further substantiation on why these variables were chosen.
5. The authors chose to use a multinomial regression model to illustrate correlates of one or more forms of interpersonal violence. However, it is unclear whether an ordinal logistic regression model was explored or feasible. Additionally, there may be different correlates of each form of violence. I suggest modeling each form of violence separately, as a sensitivity analysis, to determine whether this aggregation is feasible.

Results:

6. Knowing that older age is associated with violence in adulthood may not be informative (I see the authors recognize this, as noted in the discussion section). That said, there should be a discussion on why lifetime exposure to violence was chosen as the outcome in the multinomial regression models, and not exposure to violence in the past year, in the methods section.

Discussion:

7. To state that the correlates and patterns of interpersonal violence between men and women are different does not appear to add additional insights to the violence literature. What makes this study different from others conducted in Estonia or other countries in the region, for example?
8. The discussion on the implications of both low response rates and excessive missingness is weak. For instance, it would be helpful to know how the authors would expect the results to change if there was no missingness.
9. As the authors note, in the introduction, interpersonal violence encompasses both family/intimate partner violence and community violence. Since interpersonal violence is broad and types of violence differ between women and men, it may be useful to disaggregate the prevalence of IPV and community violence by gender and discuss these differences, so as to get a better picture of polyvictimization. However, the authors also note perpetrator was not assessed. It would be helpful to discuss this as a limitation of the work.

**Minor Issues**

Introduction:

10. What kind of violence is the authors referring to when they say, “A higher prevalence of violence is associated with a greater acceptance of violence in society…”?

Methods:

11. Are there any differences in responses for electronic vs paper questionnaires?

Results:

12. The distinction between “unknown” and “missing” in Table 2 (labeled as Table 1) is unclear. I recommend that these two categories be combined.
13. Does the variable “Biological children” refer to having one or more children? If so, I think the latter label is clearer.
14. Did the authors omit “missing” and “unknown” categories from the multinomial regression models? It is unclear whether or not this is the case.
15. Which ages are “younger” and which ages are “older”? This characterization of the variable should be consistent in Table 2 and Table 4.

Discussion:

16. Note that there are no contextual variables included in the study, yet the authors state, “the contribution of this study is identifying ***contextual*** and gender specific factor…”

General:

17. There are several instances of grammatical errors (e.g. misspellings) and awkward phrasing (e.g. “men had less often higher education”).

**Recommendation**

Based on the comments above, I recommend rejection of this paper.
