# Supplementary material for: The prevalence and patterns of exposure to interpersonal violence among men and women in Estonia
Source: PLoS One. 2020 Aug 14;15(8):e0237562. doi: 10.1371/journal.pone.0237562 (PMC7428354; doi:10.1371/journal.pone.0237562)
Supplement: Attachment — Submitted filename: Response to Reviewers.docx [file pone.0237562.s003.docx]

**Response to Reviewers**

The authors are very grateful to the editor and the reviewers for their time and the valuable comments, which help us to improve the quality of the manuscript. We have made revisions according to the comments and suggestions, the answers are presented below.

**Journal Requirements:**

**1. Please ensure that your manuscript meets PLOS ONE's style requirements, including those for file naming.**

Authors appreciate this comment and have followed the PLOS ONE style templates to meet the style requirements.

**2. Please include additional information regarding the justification for sample size in this study. How was this sample size chosen - e.g. following a power calculation.**

Authors are grateful for pointing this out. We have added the following sentences to the methods section regarding the justification of the sample sizes:

“Power analysis was carried out for both surveys in order to determine optimal sample sizes. Based on the sample size calculations, the initial sample size for EWS had to be 5233 women aged 16-44 and for EMS 4800 men aged 16–54.”

In addition to that, as both of the surveys used in this paper have published survey reports with detailed description of the methodology and both are available online, we added also the following sentence:

“More detailed descriptions concerning the methodology of the surveys can be found from the survey reports”.

**3. We note that you have included the phrase “data not shown” in your manuscript. Unfortunately, this does not meet our data sharing requirements. PLOS does not permit references to inaccessible data. We require that authors provide all relevant data within the paper, Supporting Information files, or in an acceptable, public repository. Please add a citation to support this phrase or upload the data that corresponds with these findings to a stable repository (such as Figshare or Dryad) and provide and URLs, DOIs, or accession numbers that may be used to access these data. Or, if the data are not a core part of the research being presented in your study, we ask that you remove the phrase that refers to these data.**

Authors appreciate this comment. The phrase “data not shown” was removed form the manuscript. All relevant data is presented in the manuscript.

**Reviewer #1:**

**This study explored interpersonal violence issue in an understudied population. Overall, the story is not cohesive. It took multiple approaches to investigate interpersonal violence, such as different forms of interpersonal violence, polyvictimization in adulthood, and exposure to violence during last year, while the multinomial logistic regression only tested polyvictimization in adulthood. Below, I offer some suggestions in the hope of strengthening this paper’s contribution.**

Authors are very grateful for these comments, which help us to improve the quality of the manuscript. We have taken them into account and carried out changes accordingly.

**Introduction**
**1 What is the rationale to include "different forms of interpersonal violence", "polyvictimization in adulthood", and "exposure to violence during last year" in one manuscript?**

Authors appreciate this comment. To make the paper more cohesive we have decided to leave out "exposure to violence during last year" from the manuscript.

The decision to show different forms of violence separately is associated with the definition of polyvictimization: “Polyvictimization refers to the experience of multiple victimizations of different kinds (Turner, Shattuck, Finkelhor, & Hamby, 2017)”. By showing the prevalence of different forms of interpersonal violence, the authors want to give a more in-depth overview to the readers about the formation of the “polyvictimization in adulthood” variable. However, we have changed Table 3 so that it shows the prevalence and combinations of different forms of violence in childhood, adulthood and childhood and adulthood and as previously mentioned left out “exposure to violence during last year”. By carrying out this change, we wanted to make data presented in Table 3 more connected to the following Table 4.

Authors decided to use polyvictimization in the logistic regression model (not different forms of violence separately) because we wanted to focus on determining the risk factors associated with the most serious victimization profile, as in previous studies it has been shown to have the most detrimental health effects.

**2 In the introduction, I'd like to see literature review on how childhood interpersonal violence experience will affect adulthood interpersonal violence.**

Authors are grateful for this comment. We have taken this into account and carried out changes, this topic is more thoroughly addressed in the discussion.

**3 A more detailed literature review on how to define polyvictimization is needed.**
We appreciate this comment. The definition of polyvictimization was added as follows:

“PV has been defined as experience of multiple victimizations of different kinds, not just multiple episodes of the same kind of victimization (Turner et al., 2017).”

Methods
**4 why didn't use regression with interaction term to test gender difference?**
The authors appreciate this comment. Authors chose not to use regression with interaction based on the assumption that factors associated with exposure to PVA are different among men and women and the aim of this paper was to focus on showing these differences. In addition to that, we wanted to keep the models more easily interpretable for the readers.

Discussion
5 **The key finding is those who experienced childhood violence are more likely to experience polyvictimization in adulthood, which is innovative. This result should be better interpreted in the discussion.**

Authors appreciate this comment, based on that we have added a more thorough discussion as follows:

“There are several pathways which are hypothesized to cause higher rates of revictimization, it has been shown that children exposed to PV have a reduced capacity for affect regulation in adulthood, dysfunctional behavioural patterns, lower self-esteem and higher levels of psychological distress (Briere, Hodges, & Godbout, 2010; Chan, Brownridge, Yan, Fong, & Tiwari, 2011; Dugal, Godbout, Bélanger, Hébert, & Goulet, 2018). Children exposed to abuse and neglect are at increased risk for substance abuse and criminal behavior (Briere et al., 2010; Dovran et al., 2019), which can lead both to being victimized of perpetration of violence.”

**6 P19, Para 2, "...but the strong association between PVA and exposure to violence in childhood..." it should be "...association between exposure to violence in childhood and PVA"**

The authors agree with this comment and have changed the order as suggested by the reviewer.

Table
**7 There are two Table 1 in the manuscript**

The authors are grateful for pointing out the incorrect headings of the tables and have corrected the mistake.
**8 Table 3 didn't talk to Table 4. It is better to show the frequency of no interpersonal violence, one form of interpersonal violence in adulthood, and polyvictimization in adulthood between men and women in Table 3. Also, show exposure to violence in childhood in Table 3.**

The authors appreciate this comment and have carried out changes to the Table 3. We now present in Table 3 no exposure to any form, exposure to only emotional, only physical, only sexual interpersonal violence and their combinations in childhood, adulthood and in childhood and adulthood.

**9 There are many parts in the manuscript using "violence", which is not rigorous. Violence is a very broad concept. It is better to be replaced by "interpersonal violence".**
Authors highly appreciate this comment and have carried out changes in the manuscript.

**Reviewer #2:**

**There are many typographical and grammatical errors throughout the manuscript.**

The authors are grateful to the reviewer for this comment and have checked the manuscript rigorously to correct all typographical and grammatical errors.

**1. Page 11, 3rd paragraph – The authors should ensure there description of results matches the name of variable levels in the table, e.g. “one child” should be “one biological child” and “good” should be “good or very good”.**

The authors appreciate pointing out this inconsistency in the manuscript and have changed this in the manuscript.

**2. Page 12, 1st paragraph – The authors fail to include “than women” and “than men” when making comparison throughout the manuscript.**

We appreciate this comment and have carried out changes in the manuscript accordingly. For example:
“As shown in Table 2, having higher education (24.7% *vs.* 42.6%) being officially married (27.2% *vs.* 31.8%), and having at least one biological child (43.6% *vs.* 59.4%) was less common among men compared to women. Men estimated their financial situation more often to be good or very good than women (16.7% *vs.* 10.0%)”

**3. Page 13, 1st paragraph – The authors should include the number of men and women who had been exposed to violence along with the percentage.**
The authors appreciate this comment and have added the numbers of men and women as follows:

“Two thirds (n=829, 66.6%) of men and over half (n=862, 54.2%) of women had been exposed to some form of interpersonal violence in their lifetime.”

**4. Page 14, 2nd paragraph – The authors should insert “during the last year” when describing table 3 results.**

We appreciate this comment. Table 3 has been changed and exposure to violence during last year has been omitted form the manuscript.

**5. Page 15, 1st paragraph – The authors should indicate what questionnaire was used to examine violence in childhood.**

Authors appreciate this comment. The NorVold Abuse Questionnaire was used to measure exposure to violence in childhood and adulthood. We have added information regarding that to the methods section as follows:

“In both surveys, the NorVold Abuse Questionnaire (NorAQ) was used to assess exposure to interpersonal violence in childhood and adulthood (25,26).” In addition to that, we added the answer alternatives for each situation presented in the NorAQ to the methods section: “There were four answer alternatives which were same for all questions: 1) no; 2) as a child (before the age of 18); 3) yes, as an adult (when being 18 years old or older) 4) yes, as a child and as an adult (before and after the age of 18)”.

**6. Page 15, 2nd paragraph – The authors should delete their description of significant findings for age in table 4 since they are borderline**.
The authors appreciate this comment. The age variable in Table 4 shows the change in the adjusted odds ratio per one year, which is relatively short period of time. In case we had chosen to use age groups in multinomial regression analyses, the adjusted odds ratios would have been bigger. However, we agree that in previous manuscript the description of this variable could have caused confusion and to make it clearer that AOR is presented for one year we changed the description of the variable.

**7. Table 4 – The authors should include the numbers along with the percentages of men and women who were affected by violence. The authors should include a footnote indicating the variables used for adjustment.**

Authors are grateful for this comment. Footnote indicating the variables used for adjustment and the numbers of men and women affected by one form of interpersonal violence and PVA were added to the Table 4.

**Reviewer III**

**Major Issues**

Introduction:

1. **In this paper, the authors make an implicit assumption that family/intimate partner violence and community violence can be aggregated together. A discussion of why this is feasible is missing.**

Authors highly appreciate this comment. However, we want to point out that the main objective of this paper was to give a more comprehensive picture about the overall lifetime exposure to interpersonal violence, not to evaluate exposure to violence in different situations (intimate partner versus community violence). Much of the research has focused on only one kind of victimization (Simmons, Wijma, & Swahnberg, 2015), however, this can cause the underestimation of the full burden of violence (Turner, Finkelhor, & Ormrod, 2010).

To get a better overall understanding about the total burden of interpersonal violence, the authors have decided to aggregate family/intimate partner violence and community violence. The authors agree that in the previous manuscript it was poorly explained and we have carried out changes to the introductions as follows: “Most of the research on interpersonal violence has focused on one of the types of interpersonal violence, like violence perpetrated by current of previous partner. However, many researchers have drawn attention to the fact, that leaving out other types of violence, where the perpetrator was somebody else than the intimate partner or that took place in earlier stages of life, results in lower prevalence estimates and a limited understanding about the total burden of violence (Finkelhor, Ormrod, & Turner, 2007b, 2007a; Simmons et al., 2015; Turner et al., 2010). “

Nevertheless, the authors also note the limitation of the used NorVold Abuse Questionnaire, which does not ask questions concerning the perpetrator. To address this, further discussion was added to the limitations of the paper. The authors agree that the patterns and consequences of violence, according to the perpetrator can be very different.

2. **Relatedly, it is unclear why the authors chose to distinguish the three different forms of violence – physical, sexual, and emotional (yet chose to aggregate interpersonal violence).**

We agree with this comment and we have added a more detailed discussion explaining these decisions.

The decision to keep different forms of violence separately is associated with the definition of polyvictimization, which was also added to the manuscript: “Polyvictimization refers to the experience of multiple victimizations of different kinds”(Turner, Shattuck, Finkelhor, & Hamby, 2017). The prevalence of emotional, physical and sexual violence was presented to give a better overview to the readers about the formation of the polyvictimization in adulthood variable. In addition to that, this kind of data showing the prevalence of different forms of interpersonal violence across gender on population basis has been completely lacking in Estonia.

Since in the NorVold Abuse Questionnaire there are no questions regarding the perpetrator of the lifetime violence, we were not able to present this data. This is addressed as one of the limitations of the paper.

3. **A discussion of the hypothesized prevalence of violence (e.g. based on other studies in the same region, or IPV studies in Estonia) is missing**.

Authors appreciate this comment. We have added data from previous research carried out in Estonia.

“A previous study demonstrated that in Estonia 17.2% of women had been exposed to physical and 4.1% to sexual intimate partner violence during the last year and it was an important contributor to sexual risk behaviour and adverse sexual health outcomes among women of reproductive age in Estonia (Laanpere, Ringmets, Part, & Karro, 2013). A study carried out by European Agency for Fundamental rights showed that since the age of fifteen every third woman had been exposed to physical or sexual violence by partner/non-partner (European Union Agency for Fundamental Rights, 2014).”

Methods:

4. **It is unclear why the aim is modified to “to analyze the association between PVA and variables identified in the literature to be associated with a higher risk for exposure to *IPV among women*” when the introduction sets the paper up to avoid focusing solely on IPV among women. As such, the “independent variables” sub-section needs further substantiation on why these variables were chosen.**

The authors agree with this comment. We have carried out changes to the paragraph, where we explain why these independent variables were chosen.

“The second aim was to analyze the association between exposure to one form of violence, PVA and socio-demographic variables identified in the literature to be associated with a higher risk for exposure to interpersonal violence among men and women (Nyhberg 2013, Romans 2007, Balsam 2005, Dovran 2019).”

5. **The authors chose to use a multinomial regression model to illustrate correlates of one or more forms of interpersonal violence. However, it is unclear whether an ordinal logistic regression model was explored or feasible. Additionally, there may be different correlates of each form of violence. I suggest modeling each form of violence separately, as a sensitivity analysis, to determine whether this aggregation is feasible.**

We appreciate this comment. In this paper, we have decided to focus on polyvictimization in adulthood and not each form of interpersonal violence separately. This decision was made based on previous literature showing that polyvictimization independently has the strongest effect predicting poor health outcomes. The main focus of this paper was to analyze the differences in the prevalence and patterns of interpersonal violence by gender.

Results:

6. **Knowing that older age is associated with violence in adulthood may not be informative (I see the authors recognize this, as noted in the discussion section). That said, there should be a discussion on why lifetime exposure to violence was chosen as the outcome in the multinomial regression models, and not exposure to violence in the past year, in the methods section.**

The authors appreciate this comment and considered also using the exposure to violence during last year in the multinomial regression models. However, exposure to violence during last year gives information only about a very limited time period. When assessing exposure to violence during last year or the most recent violent event, it can cause leaving out all events which took place earlier in life. The authors wanted to show in this paper more comprehensive picture about the total burden of violence during lifetime. Authors have added information to the introduction regarding why we decided to use lifetime exposure to interpersonal violence. We also want to point out, that the age structure of men and women included in the analysis was the same. Moreover, we used age as one important control variable in the model.

Discussion:

7. **To state that the correlates and patterns of interpersonal violence between men and women are different does not appear to add additional insights to the violence literature. What makes this study different from others conducted in Estonia or other countries in the region, for example?**

Authors are very grateful for pointing this out. This is the first study in Estonia and Eastern-European region analyzing the patterns of violence among men and women on a population basis. We have added further discussion regarding the differences we found between men and women in their lifetime exposure to violence.

In addition to that, research focusing on interpersonal violence polyvictimization in adulthood is very limited. This paper contributes to the violence literature by analyzing the factors associated with exposure to polyvictimization in adulthood. Further discussion was added to the manuscript.

8. **The discussion on the implications of both low response rates and excessive missingness is weak. For instance, it would be helpful to know how the authors would expect the results to change if there was no missingness**.

Authors appreciate this comment. However, we want to point out, that the questionnaires of both surveys were quite long, including more than one hundred questions, which could have affected the response rates. The questions concerning violence, which were in the end of the questionnaire, were filled in rather well (over 95% of the respondents answered to the questions concerning exposure to violence), considering the sensitivity of these questions.

To address the possible implications of the response rates, we added further discussion as follows:

“The response rates of both surveys, 47% in EWH and 44.5% in EMS were in the range expected and similar response-rate estimations were used when calculating the sample size. In addition to that, the final samples of both EWH and EMS were representative and had no selectivity bias. Previous population-based studies focusing on violence have yielded similar response rates (20,33). The prevalence of interpersonal violence in EWH survey is comparable with the results of the previous studies carried out in Estonia (20,47). However, there is a possibility that the most highly victimized men and women were not able or chose not to participate in the surveys. Based on that, it is possible, that the prevalence of PVA could be even higher than shown in this paper.”

9. **As the authors note, in the introduction, interpersonal violence encompasses both family/intimate partner violence and community violence. Since interpersonal violence is broad and types of violence differ between women and men, it may be useful to disaggregate the prevalence of IPV and community violence by gender and discuss these differences, so as to get a better picture of polyvictimization. However, the authors also note perpetrator was not assessed. It would be helpful to discuss this as a limitation of the work.**

Authors agree with comment and have added further discussion concerning the limitations associated with not knowing the perpetrator of violence

“Information concerning the perpetrator of the violence could offer better explanations regarding the differences of exposure to different forms of violence among men and women. From previous research it is known than women are more often exposed to systematic forms of intimate partner violence, which could explain higher levels of polyvictimization among them (Johnson, 2011).”

**Minor Issues**

Introduction:

10. **What kind of violence is the authors referring to when they say, “A higher prevalence of violence is associated with a greater acceptance of violence in society…”?**

Authors are grateful for pointing out this and we have carried out changes as follows:

“A higher prevalence of interpersonal violence is related to greater acceptance of violence in society, lower gender equality, and many other societal factors.”

Methods:

11. A**re there any differences in responses for electronic vs paper questionnaires?**

There were no differences, authors have added this information also to the manuscript. “There were no significant differences between the two responding methods regarding responses about exposure to violence or socio-demographic characteristics.”

Results:

12. **The distinction between “unknown” and “missing” in Table 2 (labeled as Table 1) is unclear. I recommend that these two categories be combined.**

Authors have considered combining these two, but as the “unknown” shows respondents who have answered but did not know the education of their mother/father we have decided to keep them separated. This can be interpreted as an indicator of the relationship between child and parent when they are not aware of the educational level on their parent.

13. **Does the variable “Biological children” refer to having one or more children? If so, I think the latter label is clearer.**

We appreciate this recommendation and have made changes as suggested.

14. **Did the authors omit “missing” and “unknown” categories from the multinomial regression models? It is unclear whether or not this is the case.**

Authors appreciate this comment. Missing values were omitted from the analysis. This information was added to the methods section of the manuscript.

15. **Which ages are “younger” and which ages are “older”? This characterization of the variable should be consistent in Table 2 and Table 4.**

We appreciate for pointing this out. In the multinomial logistic regression in Table 4 age was used as a discrete variable, in Table 2 age groups were presented. This was not clearly explained in the previous version of the manuscript and authors have added additional information concerning that and changed the label in Table 4.

Discussion:

16. **Note that there are no contextual variables included in the study, yet the authors state, “the contribution of this study is identifying *contextual* and gender specific factor…”**

Authors agree with this comment and have replaced “contextual” with “socio-demographic” factors.

General:

17. **There are several instances of grammatical errors (e.g. misspellings) and awkward phrasing (e.g. “men had less often higher education”).**

Authors have thoroughly checked the spelling and phrasing of the manuscript and carried out corrections.
