# Supplementary material for: The prevalence and patterns of exposure to interpersonal violence among men and women in Estonia
Source: PLoS One. 2020 Aug 14;15(8):e0237562. doi: 10.1371/journal.pone.0237562 (PMC7428354; doi:10.1371/journal.pone.0237562)
Supplement: Attachment — Submitted filename: Response to reviewer.docx [file pone.0237562.s004.docx]

**Response to Reviewers**

The authors are very grateful to the reviewers for their time and the valuable comments, which have helped us to improve the quality of the manuscript. We have made revisions according to the comments and suggestions, the answers are presented below.

**1. Throughout the paper, I am quite confused about the sample size. Based on page 8, I think the final analytical sample size is 1,244 for women and 1,590 for men. But in Table 4, sample size for men was 1,239 and for women is 1,532. Please check the sample size carefully and be consistent across tables. I also suggest authors including final sample size in the abstract.**

Authors appreciate this comment. The final sample for analysis included 1244 men and 1590 women. Only in the regression analysis respondents who did not answer some of the questions were excluded. We added additional information regarding that to the methods section under subheading “Analysis”.

*“All respondents who did not answer some of the questions in this study were excluded from the models, the responses of 1239 men and 1532 women were included to the multinomial logistic regression analysis.”*

**2. Page 4: “Exposure to four of more adverse childhood experiences…”; You mean “four or more”?**

Authors are grateful for pointing out this mistake and have corrected it.

**3. Page 13: The authors have highlighted “Two thirds (n=829, 66.6%) of men and over half (n=862, 54.2%) of women had been exposed to some form of interpersonal violence in their lifetime” as one of major findings several times in the manuscript. But they are not shown in Table 3. I suggest authors adding another column in Table 3 presenting the prevalence of EVER experiencing different forms of violence (either in childhood or adulthood).**

Authors appreciate this comment highly. We added a new column “Lifetime exposure” to the Table 3, as was suggested by the reviewer.

**4. Maybe I miss it, but percentages of experiencing only one form of violence and PVA are not consistent between Table 3 and Table 4. For example, men who experienced only one form of violence and multiple forms of violence in adulthood is 19.7 (2.8+15.9+1.0) and 2.2 (1.8+0.2+0.2) in Table 3, but it is 28.9% and 5.8% in Table 4.**
In table 4 respondents who had been exposed to any form of violence during adulthood or both adulthood and childhood were included in the new variable used in the regression analysis model. Authors agree that this was poorly explained in the methods section and we added further explanation to the methods sections as follows:

“Both, respondents who had been exposed to violence only in adulthood and those who had been exposed to violence both in childhood and adulthood were included in this variable.”

In addition to that, further explanation was added to the results section as follows:

“Among men, 28.9% (n=358) of the respondents were exposed to one form of interpersonal violence and 5.8% (n=72) to PVA in adulthood or both in childhood and adulthood, among women 17.9% (n=274) and 12.4% (n=190), accordingly.”

**5. Also in Table 4 which conduct multinomial logistic analyses, the reference group is those who do not report interpersonal violence in adulthood. Since in Table 3, authors have differentiated interpersonal violence in childhood from that in adulthood, it is helpful to include a note in Table 4 clearly indicating who is the “reference group”.**

Authors appreciate this comment. Information regarding the reference category was added to the Table 4.

**6. In multivariate analysis, exposure to violence in childhood was only measured as a dichotomous variable. Since authors are interested in comparing people who experience one form of violence versus multiple forms in adulthood, it may be also more informative to include categorical variable measuring whether they experience only one violence or multiple violence in childhood as an independent variable.**

Authors are very grateful for this suggestion and we have changed the variable “Exposure to violence during childhood” as was suggested by the reviewer and we have carried out changes in Table 4 accordingly.
In association with that, some interesting results surfaced, and changes were carried in the discussion as follows:

*“Some of the factors associated with PVA among men and women were overlapping, such as exposure to violence in childhood and older age, it should be noted that across gender exposure to violence in childhood had a graded relationship with PVA.”*

**7. I appreciate authors’ efforts to conduct the first study of interpersonal violence in Estonia, as well in East European countries. In the discussion part, the authors mention that “the results of this study…is in line with previous results from Sweden”(page 16). What about other countries you mentioned in the introduction part, such as US? Are your findings all consistent with other developed countries or is Estonia unique in some ways compared to other contexts studied in the literature? Can the results from Estonia be generalized to other East European countries or other similar social contexts?**

Authors are very grateful for this comment.

The results of this study are consistent with previous research carried out in other developed countries, which have showed differences across gender. However, most of these studies have been carried out among children or have focused on IPV. We have changed the discussion as follows:

*“Findings from prior studies carried out in other developed countries, which have looked at gender differences in the context of IPV or violence in childhood, have showed different patterns of exposure to violence across gender.”, “…which corroborates the findings of this study”* (p. 17).

The authors of this paper believe that as the recent history of Estonia and therefore many aspects of the current social and cultural norms (such as gender equality) are similar in the Eastern European region, the findings of this paper can be generalized to other countries in the region. We have added this also to the discussion section of the paper.

*“In addition to Estonia, these results are particularly useful in the Eastern European region, where until now* *research demonstrating the prevalence of interpersonal violence has been scarce.”*
